# Lysosome as a Central Hub for Rewiring PH Homeostasis in Tumors

**DOI:** 10.3390/cancers12092437

**Published:** 2020-08-27

**Authors:** Ran Chen, Marja Jäättelä, Bin Liu

**Affiliations:** 1 Cell Death and Metabolism, Center for Autophagy, Recycling and Disease, Danish Cancer Society Research Center, 2100 Copenhagen, Denmark; rach@cancer.dk; 2 Department of Cellular and Molecular Medicine, Faculty of Health Sciences, University of Copenhagen, DK-2200 Copenhagen, Denmark

**Keywords:** lysosome, pH regulation, V-ATPase, lysosomal exocytosis

## Abstract

Cancer cells generate large quantities of cytoplasmic protons as byproducts of aberrantly activated aerobic glycolysis and lactate fermentation. To avoid potentially detrimental acidification of the intracellular milieu, cancer cells activate multiple acid-removal pathways that promote cytosolic alkalization and extracellular acidification. Accumulating evidence suggests that in addition to the well-characterized ion pumps and exchangers in the plasma membrane, cancer cell lysosomes are also reprogrammed for this purpose. On the one hand, the increased expression and activity of the vacuolar-type H^+^−ATPase (V-ATPase) on the lysosomal limiting membrane combined with the larger volume of the lysosomal compartment increases the lysosomal proton storage capacity substantially. On the other hand, enhanced lysosome exocytosis enables the efficient release of lysosomal protons to the extracellular space. Together, these two steps dynamically drive proton flow from the cytosol to extracellular space. In this perspective, we provide mechanistic insight into how lysosomes contribute to the rewiring of pH homeostasis in cancer cells.

## 1. Introduction

Normal cells usually have a cytosolic pH of around 7.2 and a slightly alkaline extracellular pH (~7.3–7.4) [1]. This feature is a prerequisite for many physiological processes, such as cell growth, cell volume maintenance, and tension of muscle cells [2,3]. In contrast, cancer cells receive an enormous overload of protons derived from aerobic glycolysis followed by lactate fermentation and hydration of CO_2_. This is largely due to the frequent upregulation of several enzymes including hexokinase [4,5], phosphofructokinase [6,7], pyruvate kinase [8], lactate dehydrogenase [9], and carbonic anhydrases [10], in tumors. To cope with the resulting proton stress, cancer cells activate multiple pathways to extrude protons and to avoid the backward flow of protons, leading to intracellular alkalinization (pH > 7.2) and extracellular acidification (pH < 6.8). This phenomenon is referred to as pH gradient reversal [11]. Several plasma membrane-localized ion pumps and exchangers, such as Na^+^/H^+^ exchangers (NHEs) and monocarboxylate transporters, excrete cytoplasmic protons to maintain the reversed pH gradient [1,12]. Nevertheless, the molecular mechanism for regulating pH homeostasis in cancer cells is far more complex.

The lysosome is a membrane-bound organelle for degradations of biological macromolecules delivered by endocytic, phagocytic, and autophagic pathways [13,14]. Its lumen contains more than 60 hydrolases to execute the breakdown of macromolecules. Most of these enzymes are optimally active only in the acidic lumen of lysosomes. Lysosomal pH is kept at 4.5–5 while cytosolic pH is 7–7.5. This pH difference indicates that proton concentration in lysosomes is almost 1000 times higher than in the cytosol. Thus, the lysosome is not only a place for protein degradation but also a storage compartment of protons. As both the volume and the quantity of lysosomes are increased in cancer cells [15,16], significantly more intracellular protons are stored inside lysosomes and pH homeostasis is rewired accordingly. To further expound this perspective, we discuss in more detail how lysosomal pH is maintained, and how lysosomes of cancer cells enhance scavenging of cytoplasmic protons by V-ATPase and accelerate the disposal of luminal protons by overactivation of exocytosis.

## 2. V-ATPase Makes Lysosomes as Major Intracellular Proton Repositories

**Figure 1:**
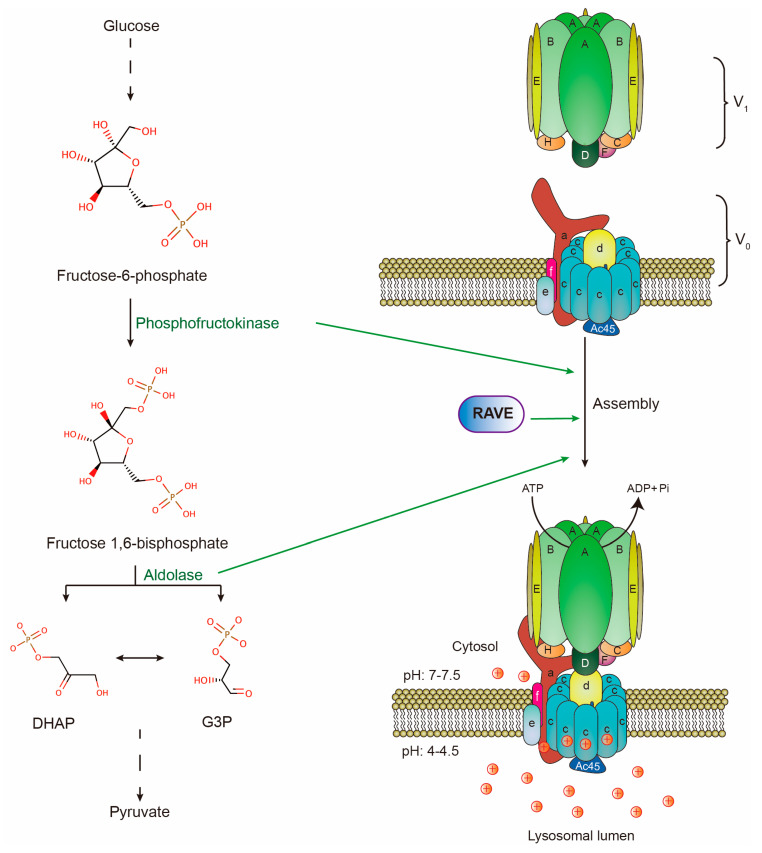
Structure of V-ATPase complex on lysosomal membranes and V-ATPase assembly machinery. Subunits A to H form V1 domain which hydrolyzes ATP. Subunits a to f form V0 domain. Protons are transported by V-ATPase against the electrochemical gradient to maintain proton equilibrium across lysosomal membranes. Two glycolytic enzymes (phosphofructokinase & aldolase) and RAVE are required for V-ATPase reassembly upon glucose readdition in yeast.

As positively charged ions, protons can cross lipid bilayers only through specific transporters and channels [17]. The proton pump on lysosomal limiting membranes is a multi-subunit complex, vacuolar-type H^+^-ATPase (V-ATPase). It is composed of a peripheral V1 domain and a membrane-embedded V0 domain. The V1 domain consists of eight distinct subunits denoted by A to H in a stoichiometry of A_3_B_3_CDE_3_FG_3_H, while the V0 domain consists of six distinct subunits denoted by a, c, c”, d, e, and f in a stoichiometry of ac_9_c”def [18,19]. Mechanistically, a heterohexamer of A and B subunits of the V1 domain hydrolyzes ATP to provide energy for the rotation of the proteolipid ring composed of subunits c and c” of the V0 domain. This rotation drives cytoplasmic protons to pass through a1 and c subunits to the lysosomal lumen [20,21]. The V-ATPase has an intra-molecular brake, H subunit, which prevents ATP-driven rotation by bridging the peripheral domain and central stalk [22]. Moreover, the reversible assembly of V0 and V1 domains regulate V-ATPase activity, thereby affecting lysosomal pH. For example, amino acid starvation promotes V-ATPase assembly and lysosomal acidification [23]. All these biochemical and structural insights have described how V-ATPase pumps protons to acidify lysosomes (Figure 1). Moreover, V-ATPase is also present in other organelles and mediates their luminal acidification, such as endosomes and synaptic vesicles [24]. However, as most of V-ATPase is delivered to lysosomal limiting membranes, the majority of cytoplasmic protons are pumped into lysosomes by V-ATPase [25,26,27,28]. Therefore, mature lysosomes become the most acidic intracellular organelles and major proton storage in cells.

## 3. pH Gradient Reversal Regulated by Lysosomal Functions

Despite their large capacity to store protons, the role of lysosomes in cellular pH regulation has been largely ignored. We introduce here cancer-associated lysosomal alterations and roll out the two ways of lysosomes to maintain cellular proton equilibrium. They are scavenging protons by V-ATPase and disposing of luminal protons by lysosomal exocytosis.

### 3.1. The Increased Proton Storage Capacity of Lysosomes

The proton storage capacity of lysosomes is determined by V-ATPase activity and lysosomal volume. In theory, either is elevated while the other is not reduced to a comparable extent would lead to increased proton storage in lysosomes and more alkaline cytosol. So far, the expression levels of V-ATPase subunits, V0/V1 assembly, and inter-molecular activations are known to regulate V-ATPase activity. Moreover, oncogenic transformation leads to lysosomal enlargement by unclear mechanisms.

#### 3.1.1. Transcriptional Regulation of V-ATPase

Because tumors undergo nutrient limitation, they have to obtain alternative energy sources and building blocks via macropinocytosis [29], and autophagy [30]. Macropinocytosis refers to the internalization and degradation of extracellular protein via a fluid phase uptake. Autophagy refers to intracellular scavenging and degradation of proteins and organelles. To meet this catabolic requirement, cancer cells need to develop compatible lysosomes. So more and larger lysosomes are fostered in tumors compared to in the normal counterpart tissues. For example, TGFbeta-1 induces lysosomal biogenesis in malignant mammary epithelial cells that lose their cell polarity and gain migratory and invasive properties [31]. Another example is that the Src-transformed mouse fibroblasts develop abnormally enlarged lysosomes [16]. A similar phenomenon is also observed during the K-RAS induced malignant transformation in breast epithelial cells [32]. How these lysosomal alterations occur is still unclear in the above conditions. However, as lysosomal pH remains constant or becomes even more acidic during oncogene-driven transformation [32,33], the increased total volume of lysosomes indeed leads to enlarged lysosomal proton storage and more alkaline cytosol [33]. On the other hand, there are a few types of tumors with enhanced lysosomal biogenesis driven by transcription factors. These transcription factors bind to the promoters of lysosomal genes, including V-ATPase components, and upregulate their expressions.

TFEB, TFE3, and MITF (MiT/TFE proteins) are master transcription factors that promote lysosomal biogenesis in normal cells of vertebrates [34,35,36]. The promoter sequences of most lysosomal genes harbor several coordinated lysosomal expression and regulation (CLEAR) consensus elements recognized by TFEB, TFE3, and MITF [34]. Some tumors upregulate expression levels of MiT/TFE proteins to boost lysosomal acidification and degradative activity. For example, pancreatic ductal adenocarcinoma (PDA) cells with overexpressed MiT/TFE proteins have much more and bigger lysosomes than normal pancreatic cells [22]. Moreover, knockdown of TFE3 in PDA cells alkalinizes lysosomes but fails to reduce lysosome volume and quantity, highlighting MiT/TFE proteins as essential regulators of V-ATPase’s transcription and activity [15]. Similarly, a higher expression level of TFEB is also observed in a few cases of ovarian [37], breast [38], and colorectal tumors [39] compared to adjacent normal tissues. There is a positive correlation between TFEB expression and pathological grade. Patients bearing tumors with higher TFEB expression often show poorer survival [37,38,39]. Genomic rearrangements and translocation also induce overexpression of MiT/TFE proteins in some rare tumors. Renal cell carcinoma (RCC) is a typical example. A small subset of RCC harbors translocation-caused TFE3/TFEB fusion, which leads to their overexpression [40]. Likewise, alveolar soft part sarcomas harbor an ASPSCR1-TFE3 fusion, leading to a chimeric protein with a stronger transcriptional activity than native TFE3 [41]. Moreover, MITF is upregulated by amplification in melanoma. Ectopic MITF expression in conjunction with the BRAF mutant can transform primary human melanocytes [42]. As target genes of MITF, 3 subunits of V-ATPase including ATP6V1G1, ATP6V1C1, and ATP6V0D2, are highly expressed in melanoma cells [43].

Besides MiT/TFE proteins, several other transcription factors regulate expression levels of V-ATPase subunits in certain types of cancers. The E2F1 is a transcription factor overexpressed in numerous cancers, including lung, breast, and hepatocellular carcinomas. Induction of E2F1 expression upregulates its direct target, ATP6V0B, which is a subunit of V-ATPase. Associated with this induction, cytosol becomes more alkaline while lysosomes become more acidic, demonstrating the important contribution of lysosomal V-ATPase to pH gradient reversal in cancer cells [44]. Another example is the overexpression of the transcription factor YY1 in gastric cancers. YY1 binds the promoter of another subunit of V-ATPase, ATP6V1A, and positively regulates expression of ATP6V1A [45]. However, there is no single subunit upregulated in all types of tumors. This may be due to the complexity of isoforms of V-ATPase subunits. Different isoforms of one subunit may prefer to assemble with different isoforms of others. Or promoters of different isoforms are controlled by tissue-specific transcription factors. 

In most tumors with aberrantly higher expression of V-ATPase subunits, disrupting pH homeostasis by inactivation or knockdown of V-ATPase inhibits either cell survival or tumor invasion. e.g., bafilomycin, a classic V-ATPase inhibitor, strongly induces apoptosis in pancreatic and gastric cancer cells [46,47]. The knockdown of V1A reduces the invasiveness of gastric cancer cells [48]. These observations highlight the importance of scavenging cytoplasmic protons by lysosomes for cancer progression.

Overall, the upregulation of V-ATPase subunits by transcription factors is an important way to promote proton uptake by lysosomes and keep the pH gradient reversed in cancer cells.

#### 3.1.2. V0/V1 Assembly 

Another mechanism of regulating V-ATPase activity is the reversible assembly of V1 and V0 domains [21,49]. In yeast, glucose deprivation triggers the disassembly of V-ATPase and lysosomal alkalinization. During this process, subunit C, as the bridge of V1 and V0, leaves the complex and separates the two domains. Then subunit H undergoes a conformational change that prevents the V1 domain from hydrolyzing ATP [50]. Conversely, the readdition of glucose induces reassembly of V-ATPase and lysosomal acidification. This process requires aldolase, phosphofructokinase, and the Regulator of ATPases of Vacuoles and Endosomes (RAVE), all of which interact with V-ATPase in a glucose-dependent manner. More importantly, RAVE facilitates the incorporation of subunit C back into V-ATPase (Figure 1) [49]. In line with the cycle of V1/V0 assembly, glucose starvation results in cytosolic acidification while glucose readdition brings the pH back to normal [51]. This strongly implies that V-ATPase assembly regulates proton equilibrium. In mammalian cells, amino acid starvation or acute glucose deprivation induces the assembly while chronic glucose deprivation triggers the disassembly of the V-ATPase complex [23,49]. DMXL2, a mammalian homolog of RAVE, may perform a conserved role in regulating V-ATPase assembly. This is suggested by the evidence that the depletion of DMXL2 in mammalian cells leads to the defect in lysosomal acidification [52]. However, the direct evidence supporting the role of DMXL2 in the maintenance of pH gradient reversal in tumors is still lacking. Reflected from the above evidence, cancer cells may promote V0/V1 assembly to maintain pH gradient reversal in two ways. One is that tumors likely employ some unknown machinery to regulate V0/V1 assembly. The other is the frequent upregulation of C subunit in tumors, such as melanoma [43], and oral squamous cell carcinoma [53], which ensures the efficient V0/V1 assembly and scavenging of protons.

#### 3.1.3. Inter-Molecular Activation

V-ATPase activity is also regulated via inter-molecular activations. STAT3, an oncoprotein, regulates V-ATPase in this way. STAT3 is a classic transcription factor overactivated in 70% solid tumors. The major way that STAT3 promotes tumorigenesis is the transcriptional regulation of target genes essential for cell proliferation, survival, angiogenesis, migration differentiation, invasion, and immunosuppression [54]. Paradoxically, a small pool of STAT3 is recruited to lysosomal limiting membranes in a coiled-coil domain-dependent manner. Moreover, STAT3 associates with and activates lysosomal V-ATPase but does not regulate V0/V1 assembly in cancer cells. More importantly, depletion of STAT3 causes lysosomal alkalinization and cytosolic acidification, demonstrating the importance of the interaction between STAT3 and V-ATPase in the maintenance of pH gradient reversal. Furthermore, acute cytosolic acidification induces additional STAT3 from nuclei to translocate to lysosomes, to counteract the stress [55]. Besides STAT3, more than 170 proteins have putative interactions with V-ATPase according to an interactome study [56]. However, only a few of them, such as chaperonin containing TCP1 subunits, SNX27, and DMXL1, are further validated by biochemical and functional experiments [56]. Therefore, it is conceivable that there may exist more other inter-molecular activations of V-ATPase to increase proton uptake of lysosomes and alkalinize cytosol. This inference is supported by the evidence that phosphofructokinase 1(PFK1) interacts with ATP6V0A4 in mouse kidney [57]. In vivo and in vitro studies reveal that disruption of this interaction severely affects proton transport and ATPase activity but not V-ATPase assembly [58]. However, this interaction and its physiological significance have never been confirmed in cancer cells. In conclusion, inter-molecular activation also plays an important role in regulating V-ATPase activity and pH homeostasis in cancer cells.

Taken together, both strengthening V-ATPase activity and enlarging lysosomal volume substantially elevate the proton storage capacity of lysosomes and alkalinize cytosol in cancer cells.

### 3.2. Lysosome-Related Pathways Are Rewired to Dispose of Protons

The lysosome can not only scavenge excessive cytoplasmic protons but also dispose of these protons by lysosomal exocytosis in cancer cells. During this exocytosis process, V-ATPase also dynamically traffics to plasma membranes, where it also pumps protons from the cytosol to the extracellular space.

#### 3.2.1. Lysosomal Exocytosis

**Figure 2:**
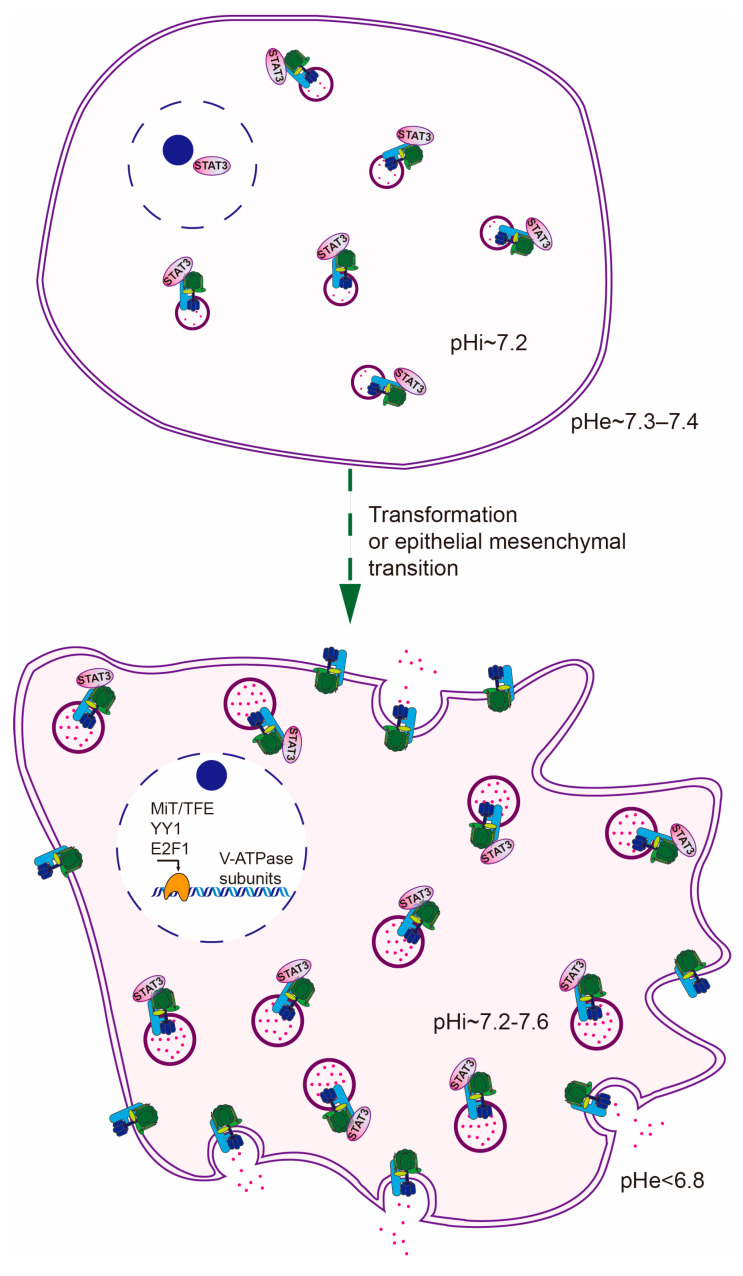
Lysosomes contribute to the maintenance of pH gradient reversal in three means: (**1**) increased proton storage capacity; (**2**) overactivated exocytosis; (**3**) plasma membrane-localized V-ATPase.

Lysosomal exocytosis is a Ca^+^ regulated process, in which lysosomes move outward to the cellular periphery and fuse with the plasma membrane, emptying their luminal protons and proteases [59]. By virtue of the dynamic feature of lysosomal exocytosis, excessive protons generated by aerobic glycolysis can continuously follow the route of lysosomes and escape from cancer cells. This rationale is reminiscent of the observation that cytosolic acidification induced outward movement of lysosomes to the peripheral region while cytosolic alkalinization did the opposite in both normal and cancer cells [60,61]. A follow-up study proved that cytosolic acidification-induced outward movement of lysosomes is en route to NHE-dependent lysosome exocytosis, which exerts an effort to preserve alkaline cytosol [62]. Moreover, enhanced lysosomal exocytosis in cancer cells is also in accord with the need for expelling chemo drugs trapped in lysosomes [63], and extracellular matrix remodeling by secreted cathepsins [64]. Empirically, increased lysosomal biogenesis always coincides with enhanced lysosomal exocytosis. For example, overexpression of MiT/TFE proteins or E2F1 not only increases lysosomal capacity but also promotes lysosomal exocytosis [44,65,66]. From the perspective of pH homeostasis, this concurrence ensures the outflow of protons in a more efficient way (Figure 2).

#### 3.2.2. Plasma Membrane-Localized V-ATPase (PM-V-ATPase)

Due to membrane trafficking, V-ATPase, as a lysosomal membrane complex, is also present on the plasma membrane [67]. This becomes more prominent in cancer cells as V-ATPase is left on or recruited to plasma membranes after lysosome exocytosis or macropinocytosis [63,66,68,69,70]. On the other hand, some cancer-preferred isoforms of V0a directly target a noticeable portion of V-ATPase to plasma membranes [71]. Noteworthy, PM-V-ATPase retains its topological direction and pumps cytoplasmic protons to extracellular space. Accordingly, artificially increasing PM-V-ATPase in mouse fibroblasts can elevate cytosolic pH from 7.0 to 7.2 and drive transformation [72]. This evidence strongly demonstrates that PM-V-ATPase is the game-changer for pH regulation, especially when the pool of PM-V-ATPase becomes bigger. In line with this, many clinicopathologically high-grade cancer cells indeed harbor abundant PM-V-ATPase [71,73,74]. The blockade of proton pump activity or knockdown of the essential membrane-integral subunit (V0a3/a4/c) can significantly acidify cytosol and inhibit invasion [71,73,74]. In contrast, overexpressing V0a3 in cancer cells could further increase cytosolic pH and invasiveness [73]. Thus, PM-V-ATPase exerts a great effect on pH gradient reversal in cancer cells.

Altogether, both lysosome exocytosis and PM-V-ATPase can facilitate cancer cells to expel protons to extracellular space, thereby alkalinizing cytosol and acidifying extracellular space.

## 4. Conclusions and Closing Remarks

Driven by oncogenic pathways and transcription factors, lysosomes, serving as major intracellular proton repositories, are reprogrammed with several fundamental alterations to accommodate excessive protons generated by aerobic glycolysis. These alterations including increased proton storage capacity, exocytotic activity, and plasma membrane-localized V-ATPase ensure efficient scavenging and expelling of protons by lysosomes. Thereby, the whole pH homeostasis is elaborately readjusted in cancer cells. Hence, we conclude the lysosome emerges as a central hub for rewiring pH homeostasis in tumors.
